# A Question of Origins: Non-neuronal Sources of Amyloid-β

**DOI:** 10.1007/s12264-025-01572-4

**Published:** 2026-01-19

**Authors:** Andrew Octavian Sasmita, Constanze Depp

**Affiliations:** 1 Department of Anatomy and Neuroscience, University College Cork, Cork, T12 XF62 Ireland; 2 APC Microbiome Ireland, University College Cork, Cork, T12 YT20 Ireland; 3 Department of Neurogenetics, Max Planck Institute for Multidisciplinary Sciences, 37075 Göttingen, Germany; 4 F. M. Kirby Neurobiology Center, Department of Neurology, Boston Children’s Hospital, Harvard Medical School, Boston, MA 02115 USA; 5 Stanley Center for Psychiatric Research, Broad Institute of MIT and Harvard, Cambridge, MA 02142 USA

**Keywords:** Alzheimer’s disease, Amyloid-β, Amyloid precursor protein, Glia, Neurons, Oligodendrocytes

## Abstract

Amyloid-β (Aβ) plaques and neurofibrillary tau tangles are hallmarks of Alzheimer’s disease (AD). While the intracellular localization of tau tangles within neurons nominates them as the primary producers of tau, the cellular origin of Aβ is less clear as plaques accumulate extracellularly. Neurons have been considered the sole source of Aβ, leading to the generation of many AD animal models expressing familial AD protein variants specifically in neurons. However, emerging evidence showed that non-neuronal cells abundantly express amyloid precursor protein (APP) and its processing machinery. Among these, oligodendrocytes (OLs) exhibit the highest expression of amyloidogenic components, produce Aβ, and contribute to plaque burden *in vivo*. Here, we highlight reports on non-neuronal Aβ production in the context of AD and the function of APP processing in these cells. Understanding Aβ processing in non-neuronal cells might enable the identification of novel therapeutic targets, especially in humans whose brain structures differ greatly from animal models.

## Introduction

Alzheimer’s disease (AD) is the most common neurodegenerative disease and the leading cause of dementia [1], wherein the predominant form of the disease is the late-onset, sporadic AD (SAD). Regardless of the age of onset, the two primary neuropathological hallmarks of AD pathology are the accumulation of extracellular amyloid-β (Aβ) plaques and intracellular phosphorylated neurofibrillary tau tangles. While their aggregation is considered pathological, both the amyloid precursor protein (APP) and the microtubule-associated protein tau are expressed throughout life and therefore likely fulfill essential physiological functions in the brain under homeostatic conditions. In this review, we first present an overview of APP processing and Aβ production, as well as the often-overlooked non-linearity between Aβ production and resulting plaque deposition. We then elaborate on current evidence that Aβ is not just produced by a singular cell type, neurons, but also by other cell types, and its potential implications for amyloid-targeting therapeutic strategies.

## Overview of Amyloidogenic Processing, Aβ Production, and Plaque Deposition

Aβ oligomerizes, fibrillates, and aggregates in Alzheimer’s disease (AD), eventually forming extracellular plaques rich in β-sheets. In this section, we cover the basis of APP processing and highlight the threshold concentration required for Aβ production for plaque deposition.

### The Amyloidogenic Pathway and Effects of Notable FAD Mutations on APP Processing

APP is the protein product of the *APP* gene situated on chromosome 21 in humans and the *App* gene on chromosome 16 in mice. The human and mouse APP proteins share approximately 97% amino acid sequence similarity with differences in specific regions, particularly in the Aβ domain (i.e., positions 5, 10, and 13), which influence amyloidogenic processing [2]. APP and its mammalian homologs, amyloid precursor-like protein 1 (APLP1) and APLP2, are single-pass transmembrane proteins characterized by a short intracellular C-terminal domain and an extensive N-terminal extracellular region, resembling the structure of a transmembrane receptor protein [3]. Among the three, however, only APP possesses the domain for Aβ. Although *App*-null mice are long-lived, double knockout of *App* and *Aplp2* is predominantly lethal [4], underscoring the importance of APP and its homologs during development.

Aβ and APP were initially discovered through the biochemical analysis of plaque content in the early 1980s. Glenner and Wong first purified cerebrovascular amyloid deposits from human autopsies and sequenced the initial 24 amino acids of a highly enriched 4 kDa protein, which they termed Aβ [5]. Subsequently, the same protein was isolated from Aβ plaque cores in AD patients [6]. The protein sequence identified was then used to develop cDNAs and locate an mRNA encoding a larger polypeptide, referred to as APP, encompassing the Aβ peptide sequence [7–9]. Further research into *APP* mutations that cause familial AD (FAD) revealed that each mutation poses varying consequences to the enzymatic cleavage of APP or Aβ fibrillization, further elaborated below.

**Fig. 1:**
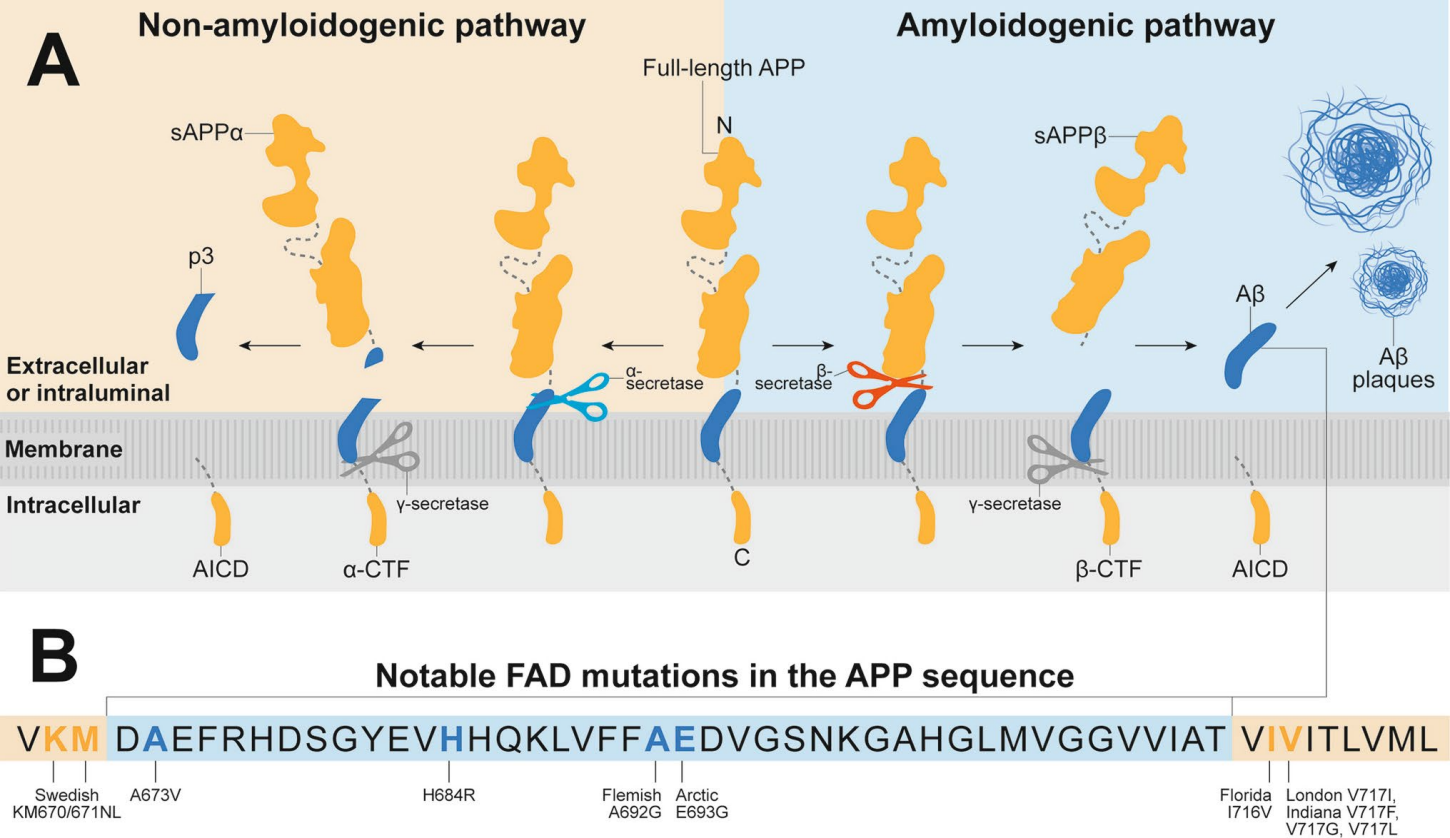
APP processing and notable FAD mutations in the APP sequence.** A** Non-amyloidogenic and amyloidogenic processing of APP. Full-length APP goes through both non-amyloidogenic and amyloidogenic processing, which starts with cleavage by α- and β-secretases (yellow and blue scissors, respectively), releasing sAPPα and sAPPβ, respectively. The second cleavage occurs via γ-secretase (gray scissors), which releases p3 in the non-amyloidogenic pathway and Aβ in the amyloidogenic pathway. **B** Location of notable FAD mutations in the APP sequence discussed throughout the review. The section of sequence highlighted in blue represents the Aβ sequence.

As the known precursor to Aβ, APP primarily undergoes one of two cleavage fates: Non-amyloidogenic and amyloidogenic processing, represented in Fig. 1A. In amyloidogenic processing [10], full-length APP is first cleaved by β-secretase (BACE) between the amino acid positions 671/672, resulting in the release of the major N-terminus portion of the APP ectodomain termed soluble APP β (sAPPβ) into the extracellular or intraluminal space. The other membrane-tethered product of BACE cleavage is the 99-amino-acid-long β-cleavage C-terminal fragments (β-CTFs) (Fig. 1A). The Swedish mutation is a well-known mutation at the β-cleavage site of APP with an immediate consequence of a significantly higher production of total Aβ [11, 12] by making APP a better substrate for BACE1. APP with this mutation undergoes a substitution of amino acids K and M to N and L at positions 670 and 671 of the APP sequence (Fig. 1B), respectively [13]. The robustness of the Swedish mutation in increasing total Aβ production also resulted in the generation of various widely used mouse models of cerebral amyloidosis containing this mutation, such as the 5xFAD and *APP*^*NLGF*^ model mice [14, 15]. Another mutation near the N-terminus of the Aβ region of APP is the A673V, which was documented to cause autosomal recessive inherited AD [16] by elevating β-cleavage of APP. As the homolog of BACE1, BACE2 is known to cleave within the Aβ region of APP (i.e., after the F residues at positions 690 and 691 of the APP sequence). BACE2 has also been shown to markedly increase Aβ production in individuals with the Flemish missense mutation (A692G) of APP [17]. Similarly situated in the middle of the Aβ region of APP, the Arctic mutation (A692G) was reported to enhance Aβ protofibril formation, which led to a reduction of plasma Aβ levels of patients carrying this mutation [18].

β-cleavage can generate Aβ at varying lengths. For instance, BACE1 cleavage can generate Aβ11-x species in the presence of H684R substitution in the human Aβ sequence, preventing the formation of full-length Aβ1-x, such as the well-known Aβ1-40 and Aβ1-42 [19]. This is further supported by the decrease in Aβ11-x generation in familial AD mutations that prefer the generation of Aβ1-x, such as the Swedish mutation [13]. In fact, the generation of N-terminally truncated Aβ species, Aβx-42, has been reported in many AD cases [20], with prominent ones including pyroglutamate Aβ3-x and Aβ4-x. The production of such truncated forms of Aβ could also be attributed to alternative cleavage enzymes such as meprin [21]. Although Aβ1-40 and Aβ1-42 are the predominant Aβ species in most mouse models of amyloidosis, N-terminally truncated Aβ is abundant in human AD samples [22, 23]. Aβ with shorter N-termini was previously reported to enhance aggregation compared to full-length Aβ species *in vitro* [24]. In line with the reduction of Aβ42 in the CSF of AD patients, levels of N-terminally truncated Aβ species were also reported to be reduced [25, 26]. *In vivo*, transgenic mice expressing Aβ4-42 were shown to accumulate plaques at high propensity and stability with concomitant behavioral deficits [27]. Despite the interest in N-terminally truncated Aβ, the predominant Aβ species in human AD, be it SAD or FAD, are still Aβ1-40 and Aβ1-42 [22].

Finally, γ-cleavage, primarily between the amino acid positions 711/712 and 713/714 of APP, generates Aβx-40 and Aβx-42, respectively. This cleavage releases Aβ to the extracellular or intraluminal space while canonically leaving the APP intracellular domain (AICD) membrane-tethered. This cleavage process is mediated by γ-secretase, a multi-subunit protease complex consisting of presenilin (PSEN), nicastrin (NCT), anterior pharynx-defective 1 (APH-1), and presenilin enhancer 2 (PEN-2). Of these components, PSEN is known to be the catalytic subunit of γ-secretase, with PSEN1 exhibiting higher expression in the brain and Aβ-producing capacity than PSEN2. Much like BACE, γ-secretase can cleave the β-CTF at varying sites and generate Aβ at varying lengths, with the most prolific being Aβ40 and Aβ42. Although γ-secretase also serves as the final cleavage enzyme in the non-amyloidogenic pathway, it was shown *in vitro* that the propensity of γ-cleavage to yield Aβ is higher than its role in producing p3 in the non-amyloidogenic pathway [28]. The various FAD mutations distal to the γ-cleavage site of APP induce a preference for Aβ42 generation, leading to a higher Aβ42/40 ratio. Substitution of amino acid 717 of the APP sequence from V to I (London) [29], F (Indiana) [30], G [31], or L [32] was shown to cause FAD via modulation of γ-secretase cleavage. Similarly, the I716V mutation (Florida) increases γ-cleavage and increases Aβ42 and Aβ43 production, which synergizes with V717I when both mutations are present [33].

In view of their predominant roles as secretases, it is important to note that APP is not the sole substrate of both BACE1 [34] and γ-secretase [35]. For example, neuregulin 1—which regulates myelination—is a cleavage substrate of both BACE1 and γ-secretase [36]. The physiological functions of BACE and γ-secretase are further highlighted by the failure of various AD clinical trials exploring the feasibility of β- and γ-secretase inhibitors [37, 38], primarily due to adverse effects, which included hippocampal shrinkage in the case of the BACE inhibitor, verubecestat [39]. One study also reported that genetic ablation of *Bace1* resulted in impairments of synaptic vesicle release [40]. Meanwhile, a γ-secretase inhibitor was shown to block Notch signaling [41], potentially affecting cell fate in molecular circuits. Although secretase inhibitors are direct strategies to mitigate Aβ production, global inhibition of both β- and γ-secretase as a therapeutic avenue in AD came with various hurdles. Additionally, the timing of drug administration often hinges on fundamental kinetics of Aβ accumulation in the central nervous system (CNS) and should be further considered.

### Localization of Aβ in the Endolysosomal Pathway

In neurons, the trans-Golgi network (TGN) may represent the earliest site compatible with Aβ production [42, 43]. Endosomes are additionally considered a significant hub for Aβ generation due to their relatively low pH within the secretory pathway [44]. APP is thought to undergo β-cleavage in the lumen of endosomes, and β- but not α-cleavage of APP was shown to be dependent on endocytosis [45]. The maturation of endosomes is dictated not only by the acidification of the lumen by V-ATPases [46] but also by significant changes in membrane proteins and lipids, as well as varying endosomal content. Endolysosomal disruption is critical in AD and has been shown to precede significant accumulation of protein aggregates [47, 48]. Hence, we provide a brief overview of the intracellular compartmentalization of Aβ with implications for its extracellular release, a topic reviewed by others [49–52].

Endocytosis of APP and its secretases and the formation of the early endosome occur in both a clathrin-dependent and independent manner, which may differ between neurons and non-neuronal cells [53]. Internalized cargoes are sorted to be either recycled back to the surface via recycling endosomes, additionally sorted into the TGN via retromers, or, upon endosomal maturation, degraded via the fusion of late endosomes with lysosomes. Some endosomes that mature undergo invaginations of their membrane, pinching off to form intraluminal vesicles (ILVs) inside the endosomal lumen, resulting in the formation of multivesicular bodies (MVBs) [54]. Interestingly, ILVs may undergo retrofusion—a process by which ILVs fuse with the limiting membrane of MVBs to release their intraluminal cargoes into the cytosol [55]. If ILVs contain Aβ [56], it remains a possibility that retrofusion of ILVs releases Aβ into the cytoplasm and contributes to intracellular accumulation of Aβ, which has been shown to cause neuronal vulnerability even in early Braak stages [57]. Additionally, ILVs can be routed for extracellular release as exosomes upon the fusion of MVBs with the plasma membrane. Although debated, some studies have shown that Aβ in the endosomal lumen can be sequestered by glycosphingolipids on the outer membrane of ILVs, the precursor to exosomes [58–60]. The importance of exosomes in Aβ release was shown by several studies reporting that exosomes are enriched in Aβ in comparison to brain homogenates [56, 61]. Aside from permitting release of Aβ with implications for its clearance and extracellular plaque deposition [60], exosomes can mediate the propagation of Aβ between neural cells [62].

Endosomal cargoes that are not targeted for recycling or release are then targeted for degradation by fusion with lysosomes. Lysosomes, which are acidic and more abundant in the neuronal soma compared to axons and dendrites [63], play a critical role in the secretory pathway and exhibit a significantly more acidic lumen than the TGN and endosomes. Lysosomes found in the distal axon were shown to be less acidic than proximal axons [64], while the most acidic lysosomes are found in the perinuclear region of neuronal somata [65]. A study showed that APP, normally requiring endocytosis from the plasma membrane for Aβ processing, can, in fact, be directly transported to the lysosome via adapter protein 3 for Aβ processing [66]. Importantly for β-cleavage of APP, BACE1 activity was reported to be higher in a narrow acidic pH range, with peak enzymatic activity detected at pH 4.0 [67, 68], which is in the range of lysosomal pH [69]. In line with studies investigating the intracellular localization of β-cleavage, Tsang and colleagues observed that in iPSC neurons containing the APP Swedish mutation, Aβ is primarily localized in neuronal lysosomes and released via lysosomal exocytosis into the extracellular space [70]. This corroborates findings in other disease models wherein aggregation-prone proteins such as α-synuclein are also expelled into the extracellular space via lysosomal exocytosis [71]. Although Tsang and colleagues only reported lysosomal exocytotic events in the neuronal soma *in vitro*, a study proposed that under pathological conditions, axons—normally low in lysosomal content—may lose their ability to degrade accumulated structures [72]. This is particularly evident in plaque-associated axonal swellings, which harbor major lysosomal accumulations in AD models [73] and in human AD brains [74, 75]. The low number of axonal lysosomes in physiological conditions contrasts with the high lysosomal content in plaque-related axonal swellings, suggesting transport failures caused by nearby Aβ plaques, which may in turn fuel local Aβ production within swellings that are rich in lysosomes and autophagic vacuoles, further contributing to local plaque seeding via lysosomal exocytosis.

Interestingly, by labeling different endocytic maturation markers, Lie and colleagues proposed that not all structures accumulated within axonal swellings are lysosomes. Instead, they might also be autophagic vesicles or late endosomes that are less capable of content degradation [72] and further contribute to Aβ accumulation [76]. High endolysosomal Aβ concentration has been shown to promote the formation of even more Aβ oligomers with downstream consequences, which include impaired endolysosomal sorting as well as tau missorting [77–79]. In concordance with these data, a recent study elegantly characterized the proteome of plaque-associated axonal swellings found in postmortem human AD samples and reported pathways positively correlated with the formation and growth of these structures: Proteolysis dysfunction, cytoskeletal dysregulation, as well as upregulated lipid transport and metabolism [74]. In concordance with previous data, the study by Cai and colleagues noted an enrichment of proteins associated with lysosomal acidification and dysfunction [74], suggesting diminished Aβ degradation.

Lastly, several studies highlighted how modifications that influence the localization of amyloidogenic components can enhance Aβ production. *In vitro* redirection of BACE1 recycling towards the TGN has been shown to increase Aβ production [80]. Similarly, retaining endogenous BACE1 and APP within the TGN *in vitro* was found to elevate the production of both Aβ42 and Aβ40 [43]. While this remains a topic of debate, current literature suggests that BACE1 and APP partitioning may limit β-cleavage, even in the absence of familial APP mutations [81]. Due to age-related alterations in the secretory pathway, this partition might deteriorate, permitting increased β-cleavage and raising baseline Aβ levels.

**Fig. 2:**
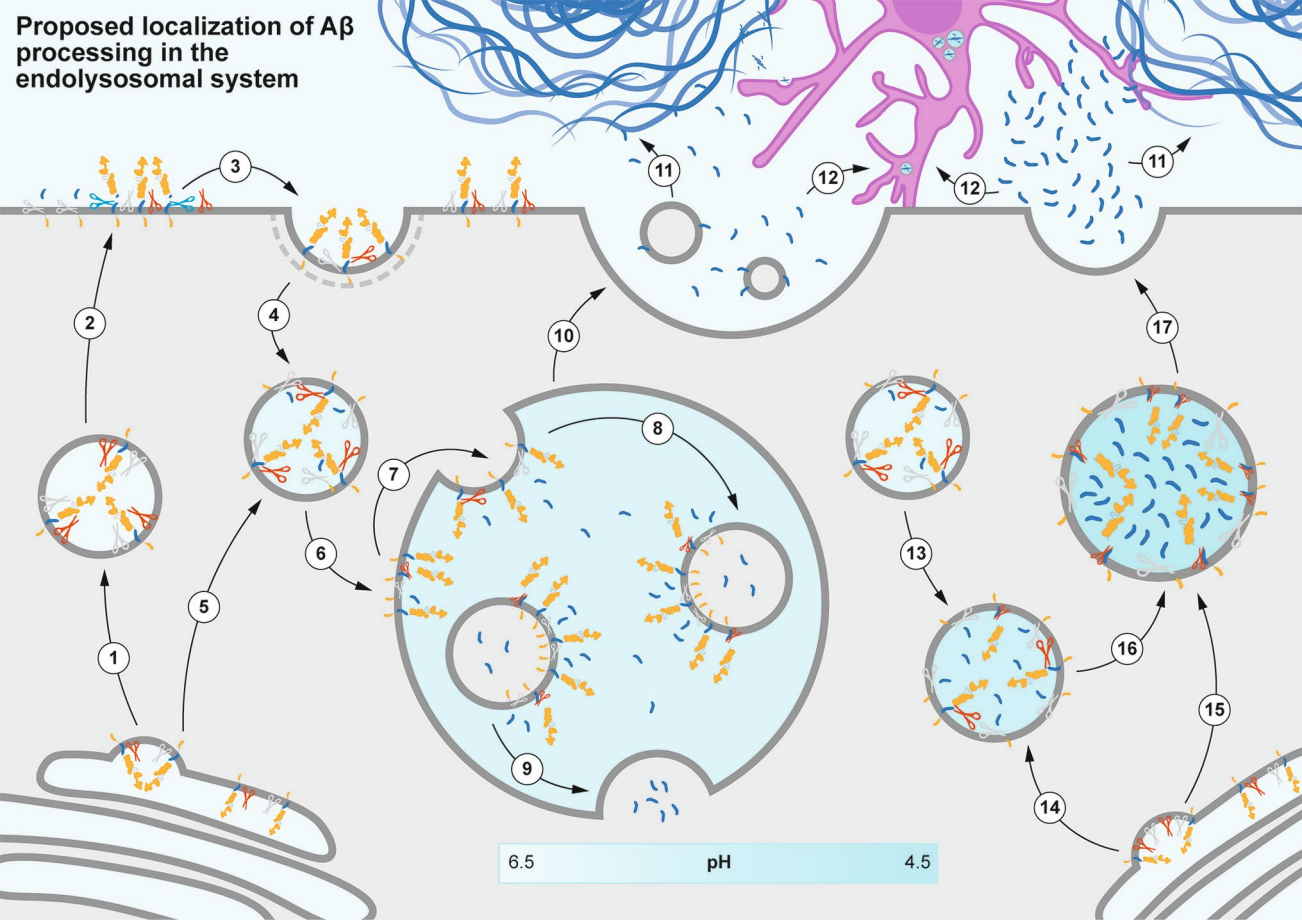
Proposed localization of Aβ processing and release in the endolysosomal system. The endolysosomal compartments are annotated in the schematic, while the different events are elaborated as follows: **1** APP and its secretases are packed into a transport vesicle upon budding from the TGN. **2** Fusion of the transport vesicles allows for APP and its secretases to be situated at the plasma membrane, wherein APP may undergo non-amyloidogenic processing. **3** APP gets endocytosed in both a clathrin-dependent and -independent manner. **4** Formation of the early endosome, wherein Aβ processing may already occur. **5** Additional APP and secretases may be directly shuttled from the TGN to early endosomes. **6** Some maturing endosomes become MVBs. **7** Invagination of the MVB limiting membrane generates ILVs. **8** Aβ produced by ILVs gets predominantly deposited in the MVB lumen. **9** If Aβ also exists within the ILV lumen, retrofusion of the ILV with the MVB limiting membrane may release Aβ into the cytoplasm, contributing to intracellular Aβ accumulation. **10** Fusion of the MVB with the plasma membrane releases its intraluminal content, which includes exosomes and Aβ, into the extracellular space, where it may be taken up by neighboring cells. **11** Other early endosomes mature to become late endosomes without ILVs. **12** APP and its secretases may be supplied from the TGN to late endosomes. **13** APP and its secretases may also be transported directly to lysosomes. **14** As late endosomes mature to become or merge with lysosomes, the intraluminal pH further decreases, which increases BACE activity and Aβ processing. **15** Lysosomal contents not targeted for degradation may be exocytosed. **16** Extracellular Aβ may act as initial plaque seeds or be sequestered by existing plaques for volumetric growth. **17** Microglia uptake Aβ and aggregate it with APOE in their endolysosomal system. **18** Compacted and fibrillar Aβ is released by microglia, allowing further plaque deposition.

Together, the experimental data discussed in this sub-section suggest that Aβ processing occurs ubiquitously throughout the endolysosomal pathway in neurons and potentially non-neuronal cells. The proposed processing machinery and Aβ localization are highlighted in Fig. 2. Specifically, for neurons, upon extracellular deposition, Aβ plaques may disrupt nearby axonal projections, resulting in axonal swellings that accumulate amyloidogenic processing components. This could lead to the local production and release of Aβ via exocytosis, which may in turn be used for plaque growth. More thorough investigations into how Aβ is released into the extracellular space from both neurons and non-neuronal cells are key in further understanding early events in Aβ plaque formation with implications for anti-Aβ therapeutic strategies.

### Non-Linearity Between Aβ Production and Plaque Deposition

**Fig. 3:**
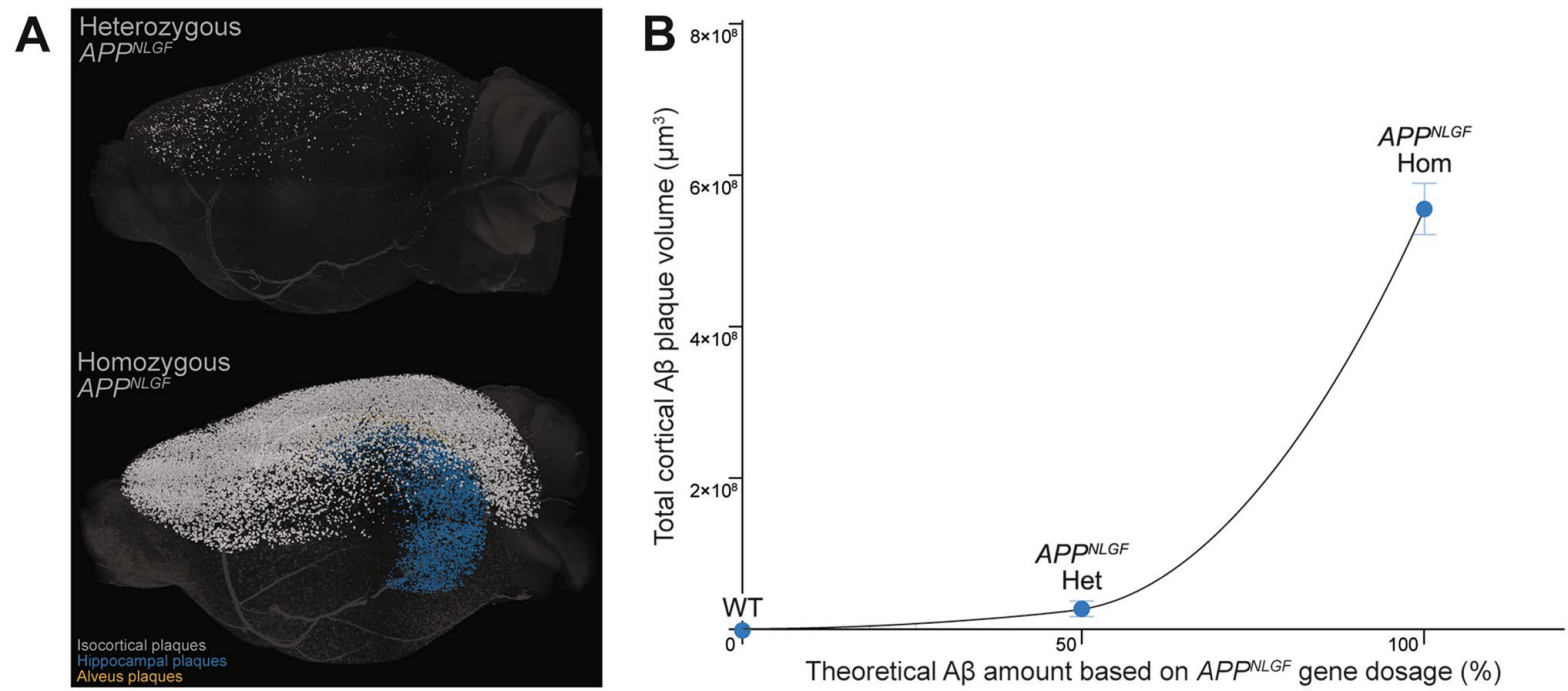
Non-linearity between Aβ production and resulting plaque deposition. **A** Representative 3D light-sheet microscopy rendering of Aβ plaque-labeled heterozygous (top) and homozygous (bottom) *APP*^*NLGF*^ mouse hemibrains. Surface volumes of plaques are rendered and visualized in different colors depending on the brain region (white-isocortex, blue-hippocampus, yellow-alveus). **B** By comparing heterozygous and homozygous *APP*^*NLGF*^ mice, which theoretically produce 50% and 100% of Aβ, respectively, at a given time point, the non-linear relationship between Aβ production and plaque accumulation can be visualized by heterozygous *APP*^*NLGF*^ mice only developing <10% of plaques seen in homozygous *APP*^*NLGF*^ mice. Adapted from [82].

Based on experimental rodent findings, the amount of Aβ produced does not scale linearly to the number of parenchymal plaques produced. This is demonstrated by heterozygous *APP*^*NLGF*^ mice, which express 50% of the mutated and humanized *APP* gene dosage, only developing less than 10% of the Aβ plaque load seen in homozygous *APP*^*NLGF*^ mice at the same time point, as highlighted in Fig. 3 [82]. Taking this into consideration, for example, a 20% reduction of Aβ plaques must mean a <20% reduction of total aggregation-prone Aβ production. In view of the sigmoidal growth kinetics of Aβ plaques observed within *in vivo* studies [83, 84], Aβ plaques seem to only manifest once a certain threshold parenchymal Aβ concentration is surpassed and significant volumetric growth occurs at high Aβ concentration. Instead of Aβ plaques, soluble Aβ concentration may be better suited to estimate steady-state Aβ production. Of note, various aspects such as age-related changes to tissue integrity and differing Aβ clearance rates in different brain regions must also be considered when quantifying Aβ generation from a specific cell type, for example, which requires further investigation.

Another argument that supports this notion is the time it takes for Aβ plaque pathology to materialize from the onset of expression of mutated APP *in vivo*. Simply put, in various AD models with FAD mutations on APP or PSEN1, not one has ever been reported to develop major plaque deposition immediately after birth. As an example, the 5xFAD mouse model, which has its transgene expression driven by the Thy1 promoter—which was reported to be active in the CNS as early as embryonic day 11 [85]—only starts exhibiting plaque deposition starting 1-2 months [14]. This highlights time as an essential component for soluble Aβ to reach its threshold concentration for plaque deposition. Upon reaching said threshold concentration, major plaque seeding occurs until the number of new Aβ plaque seeds slowly plateaus while existing plaques continuously grow in volume. Ideally, primary readouts of secretase inhibitors and modulators, for example, should include measures of cerebral Aβ concentration and not just proxy measures for Aβ plaques, such is the case with Aβ positron emission tomography (PET) scans [86].

This non-linear phenomenon could, in fact, explain the struggles with Aβ therapeutic strategies, which often stem from finding the right timing of drug administration. When clinical symptoms manifest, the therapeutic window of various amyloid-targeting therapies is often viewed to be closed for preventative intervention. When viewing such therapeutic avenues as preventative, however, the question remains: How early and for how long should amyloid-targeting therapy be administered to elicit efficient preventative effects [87]? Fundamentally, basic kinetics of Aβ deposition into plaques should be considered, and preventative strategies could benefit from the administration of drugs at a much earlier time point than the onset of clinical symptoms, which would require earlier and robust diagnostic tools.

## Neurons are the Primary but not the Sole Producers of Aβ

A long-standing consensus in the field of AD remains that Aβ is primarily a neuronally derived protein, which contributes to a neuronal-centric view of AD despite mounting evidence showing that AD pathophysiology involves more than just neurons. For example, *APOE4*, the strongest risk factor gene for SAD, is a variant of the *APOE* gene highly expressed by astrocytes, and APOE4 astrocytes heftily secrete cholesterol, which promotes Aβ plaque formation [88]. Furthermore, APOE is also upregulated in disease-associated microglia (DAM) in the vicinity of Aβ plaques [89], with APOE4 microglia shown to exhibit impaired lipid metabolism [90, 91]. The TREM2 R47H mutation is another genetic risk factor for SAD [92] and has been shown to alter microglial functions in response to Aβ pathology [93] and in synaptic maintenance [94]. With increasing evidence of the involvement of non-neuronal cells in AD pathogenesis, the question thus remained: Are neurons truly the only cell type capable of yielding a significant and pathological amount of Aβ that contributes to cerebral amyloidosis?

Early work by Kang and colleagues showed that APP resembles a cell surface receptor, especially abundant in neurons [8]. This was further confirmed by the efficient production of neuronal Aβ in transgenic APP mice [95, 96]. By assessing organotypic hippocampal slices of transgenic APPSwe mice, Kamenetz and colleagues showed that the amount of Aβ production is correlated to neuronal activity, which the authors modulated by pharmacological agents [97]. The series of seminal studies further cemented the notion that only neurons produce Aβ and, in turn, led to the development of various transgenic AD mouse models that leverage the overexpression of mutant, human APP or PSEN transgenes driven by promoters, such as Thy1, which are active in a subset of excitatory neurons. Some of the most notable transgenic models with the Thy1 promoter include the 5xFAD [14], APP/PS1 [98], and 3xTg [99]. Although these models are robust in generating Aβ and plaques, pathological APP and amyloid variants are exclusively derived from targeted neurons [85].

Excitatory neurons are indeed the main producers of Aβ *in vivo* [82, 100]. This was shown by the almost complete abolishment of cerebral Aβ plaque pathology upon selectively silencing *Bace1* in dorsal telencephalic excitatory neurons via *Thy1-Cre/ERT2* [100] or *Nex-Cre* [82] in *APP*^*NLGF*^ mice at 4 and 6 months, respectively. Considering the non-linearity between Aβ production and plaque deposition, our observation that *Bace1* deletion in dorsal telencephalic excitatory neurons reduced cerebral plaque load by >95% cannot be directly interpreted as excitatory neurons producing ~95% of the total cerebral Aβ. Extrapolating values from soluble Aβ measurements instead, dorsal telencephalic excitatory neurons may, in fact, produce 80-85% of total Aβ at 6 months *in vivo* [82]. It is important to note here that analysis of *Nex-Cre Bace1*^*fl/fl*^* APP*^*NLGF*^ (*ExN-Bace1*^*cKO*^*;AD*) mice at 1 year revealed abundant cerebral plaque pathology without the excitatory neuronal contribution to Aβ burden, further highlighting excitatory neurons as the primary but not sole contributors to Aβ plaque load. Why do excitatory neurons seem to produce Aβ at an especially high rate? This might be explained by—but not limited to—excitatory neurons’ abundance, high activity, and large volume, especially when accounting for their cell bodies, dendrites, and long axonal projections [97, 101].

Ultimately, although Aβ processing has been extensively studied in neurons, whether they are the sole producers of Aβ—and by proxy the most negatively impacted by Aβ—remained largely unanswered. Veeraraghavalu and colleagues reported that transgenic APPSwe mice lacking forebrain excitatory neuronal PS1ΔE9 produced comparable levels of Aβ compared to controls at 10-12 months [102], further hinting at the contribution of Aβ from other non-neuronal cells, and mirrors our experimental findings in 1-year-old *ExN-Bace1*^*cKO*^*;AD* mice [82]. It is important to note here that the transgene in APPSwe is driven by the mouse prion promoter [103], allowing for transgene expression in non-neuronal cell types. In fact, it was only recently reported that GABAergic interneurons contribute to Aβ plaque load *in vivo* [104]. The abundance of APP processing in non-neuronal cells begs the question of whether other neural or non-neural cells could, in fact, generate considerable amounts of Aβ.

## Non-Neuronal Sources of Aβ

**Fig. 4:**
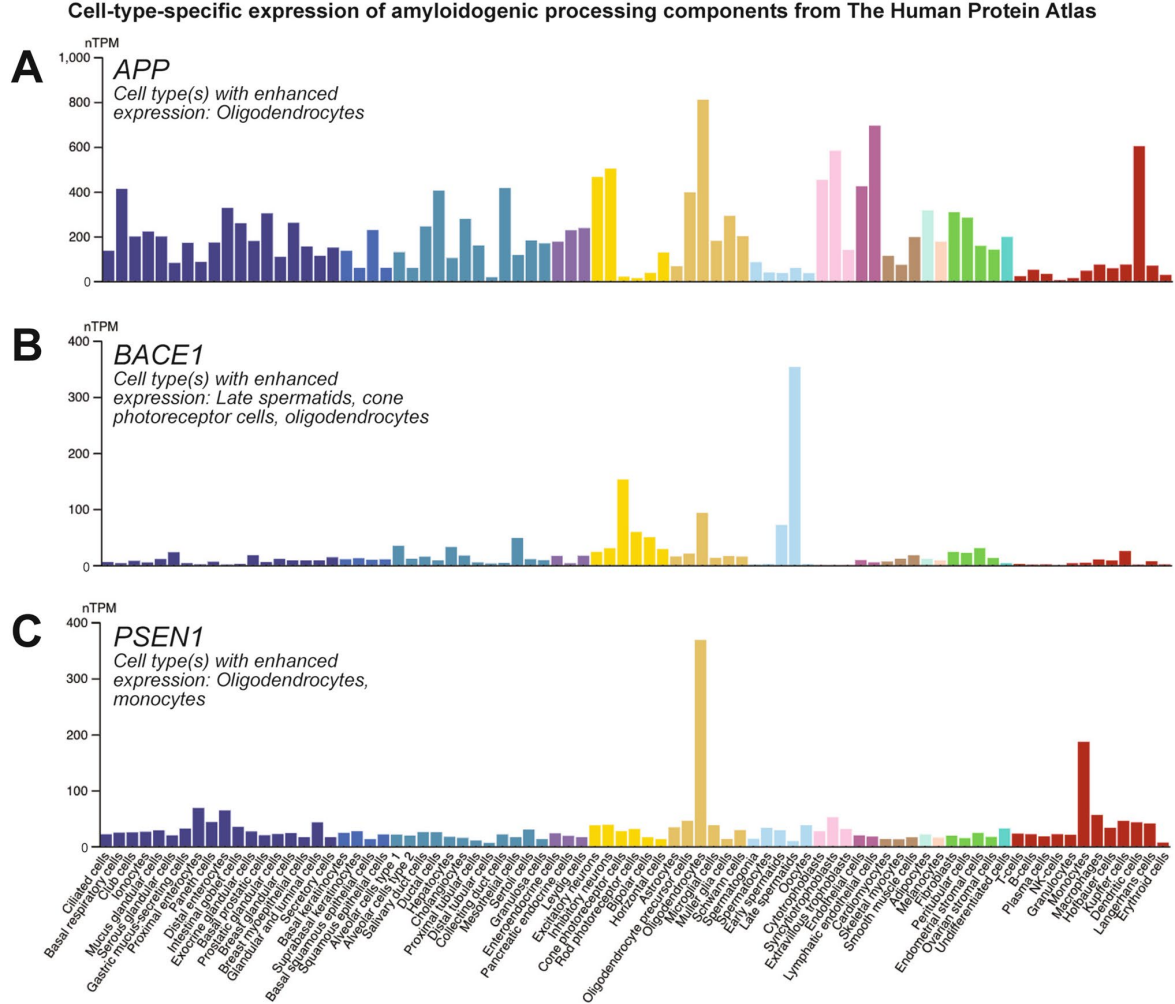
Various cell types express amyloidogenic processing components. Cell-type-specific expression of **A** *APP,* **B** *BACE1,* and **C** *PSEN1* in human tissue. Notably, OLs are noted as the cell type enriched for all 3 components. Image credit: The Human Protein Atlas, www.proteinatlas.org/, [107]. Images available at the following URL: (v24.proteinatlas.org/ENSG00000142192-APP/single+cell, v24.proteinatlas.org/ENSG00000186318-BACE1/single+cell, v24.proteinatlas.org/ENSG00000080815-PSEN1/single+cell).

Aβ is the amyloidogenic cleavage product of the larger APP, which is heavily expressed by various neural cell types [82, 105]. Earlier work, however, showed the more efficient Aβ production in neurons compared to microglia and astrocytes [106]. Despite this, APP and its processing enzymes are abundantly expressed by non-neuronal cells in various human tissues, shown in the publicly available single-cell RNA data repository, The Human Protein Atlas [107] (Fig. 4). While *APP* is abundantly expressed by various cell types (Fig. 4A), *BACE1* is especially enriched in late spermatids, cone photoreceptor cells, and oligodendrocytes (OLs) (Fig. 4B). Interestingly, *PSEN1* is enriched in OLs and monocytes (Fig. 4C). Notably, Karlsson and colleagues determined the enrichment threshold of a specific cell type as a four-fold higher expression compared to the mean of all other cell types [107].

Aβ production of other non-neuronal cells in the CNS has been reported—at least *in vitro*—from OLs, astrocytes, microglia, and endothelial cells (ECs). Of note, early work by Joachim and colleagues showed that Aβ deposits can be observed in other peripheral organs such as the skin, muscle, and intestine [108]. Aβ can also be found in the liver and the kidney, which are known to be sites of Aβ clearance [109]. Hence, investigations into which cell types throughout the body produce Aβ are complicated not only by the complex border between the nervous system and other bodily systems, but also by the far-reaching communication between different systems, and whether Aβ is present in specific organs as a terminal destination for deposition or for clearance.

One study showed expression of the APP processing machinery in wild-type mice as well as Aβ production in the gut of APP/PS1 mice [110], although the source of gut Aβ could be CNS neuronal projections that target the gut, enteric neurons, or enteric glia. Similar to reports on the transmission of gut α-synuclein to the brain, a study reported that injection of Aβ and tau N638 fibrils into the colon wall of wild-type mice resulted in the propagation of both pathologies 3 months post-injection via the vagus nerve [111]. The gut-to-brain transmission of fibrils was further blunted upon vagotomy, underscoring the potential necessity of the vagus nerve for the spreading of pathogenic protein aggregates. Upon Aβ or tau fibril injection into the colon, C/EBPβ and δ‐secretase were prominently increased in the brain [111], suggesting a shared inflamed gut-brain phenotype. This also highlights the importance of the gut-brain axis in pathological protein propagation [112]. Although neither study conclusively answers whether cell types of the gut can produce Aβ locally, single-cell transcriptomic characterization of the mouse intestinal epithelium revealed substantial *App* expression in various cell types. Of these cell types, goblet cells—the mucus-secreting cells in the gut to protect the intestinal lining and closely interact and sculpt the gut microbiome composition—exhibit *Bace2* expression higher than other gut cell types [113], which may suggest differential APP processing between intestinal cell types. Aβ in the gut may, in fact, co-aggregate with gut microbiome-derived biofilm amyloids, as is the case with α-synuclein, which induces neuropathology [114].

Similar to Aβ in the gut, another study reported that Aβ can be found in the temporalis skeletal muscle of AD patients compared to control patients [115], but whether muscle cells themselves produce Aβ to deposit locally or if Aβ found in the skeletal muscle is a byproduct of neuronal projections remains a question. The same conundrum applies to Aβ deposits seen in human skin samples, as it may be a result of Aβ generation from neuronal projections or alternatively by skin fibroblasts [12]. Also associated with the blood-brain barrier (BBB), one study reported the production of Aβ in iPSC-derived pericyte-like cells that express the Swedish APP mutation, albeit producing 100-fold lower Aβ40 and Aβ42 compared to iPSC-derived neurons also carrying the Swedish APP mutation [116]. If pericytes produce Aβ, they might contribute to BBB defects and cerebral amyloid angiopathy (CAA) in AD.

Of note, the abundant expression of amyloidogenic processing components (i.e., APP, BACE, γ-secretase) and *in vitro* evidence of Aβ production do not warrant production of Aβ and contribution to plaque deposition *in vivo* from that particular cell type or in human AD cases. *In vitro* studies that report a cell type’s ability to produce Aβ were often done in monoculture systems or in the presence of stimulating factors or inflammatory mediators [117]. Secondly, although Aβ fibrillization has consistently been reported *in vitro*, plaque deposition only occurs significantly *in vivo,* as it seems to require a multitude of factors. These factors may include the heterogeneity and communication between cell types, as well as extracellular matrix components [118, 119], which cannot be robustly recapitulated *in vitro*. It also remains intriguing why Aβ plaque formation is concentrated in the brain, especially if cell types outside the CNS express the necessary components to produce Aβ. Ultimately, non-neuronal Aβ production should be explored, but also further disentangled from the role of some of these cell types in Aβ clearance or plaque seeding [120].

**Fig. 5:**
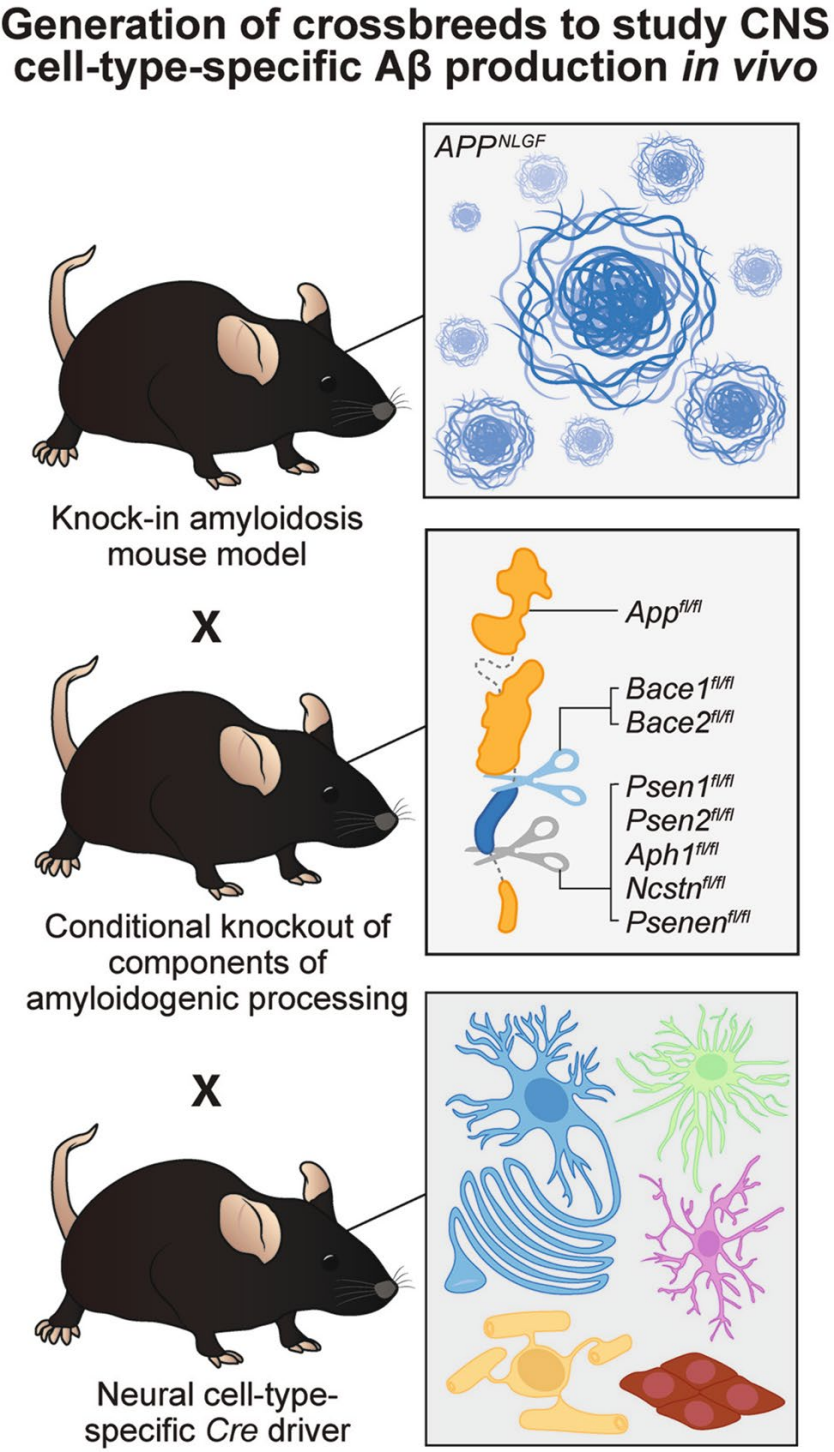
*In vivo* strategies to investigate which major CNS cell types produce Aβ and contribute to plaque burden. Cell-type-specific deletion of key amyloidogenic genes, including *Bace1*, γ-secretase subunits, and *App*, could be leveraged to explore the contribution of various non-neuronal cells to total Aβ burden. In the bottom panel, the cell type-color annotations are as follows: neurons-blue, oligodendrocytes-yellow, astrocytes-green, microglia-magenta, endothelial cells-red.

Notably, confirmatory *in vivo* studies benefited from the knock-in *APP*^*NLGF*^ mouse model of amyloidosis. In this model, endogenous APP levels are maintained by the murine *App* promoter, and APP expression is not driven solely in neurons [15], allowing for the investigation of cell-type-specific contributions to Aβ load via selective knockout approaches. For a cell type to produce Aβ, the canonical amyloidogenic components must be present, which include APP, BACE, and PSEN, the latter being the catalytic subunit of γ-secretase. Hence, strategies to study cell-type-specific contributions to Aβ load could employ crossbreeds of an AD mouse model with pan-cell-type expression of mutant, human APP, such as the *APP*^*NLGF*^ [15]. By utilizing the Cre-LoxP system, these AD mice could be crossed with mice harboring cell-type-specific deletion of either the rate-limiting enzyme gene, *Bace1* [121], or genes of the γ-secretase subunits, which include *Psen1* [122], *Aph1* [123], *Ncstn* [124], and *Psenen* [125], when crossed with mice with cell-type-specific *Cre* drivers of the CNS (Fig. 5) or in peripheral systems. Alternatively, LoxP sites could be introduced in the *App* locus of the *APP*^*NLGF*^ mice to abolish the amyloidogenic substrate production with the appropriate *Cre* drivers (Fig. 5).

**Fig. 6:**
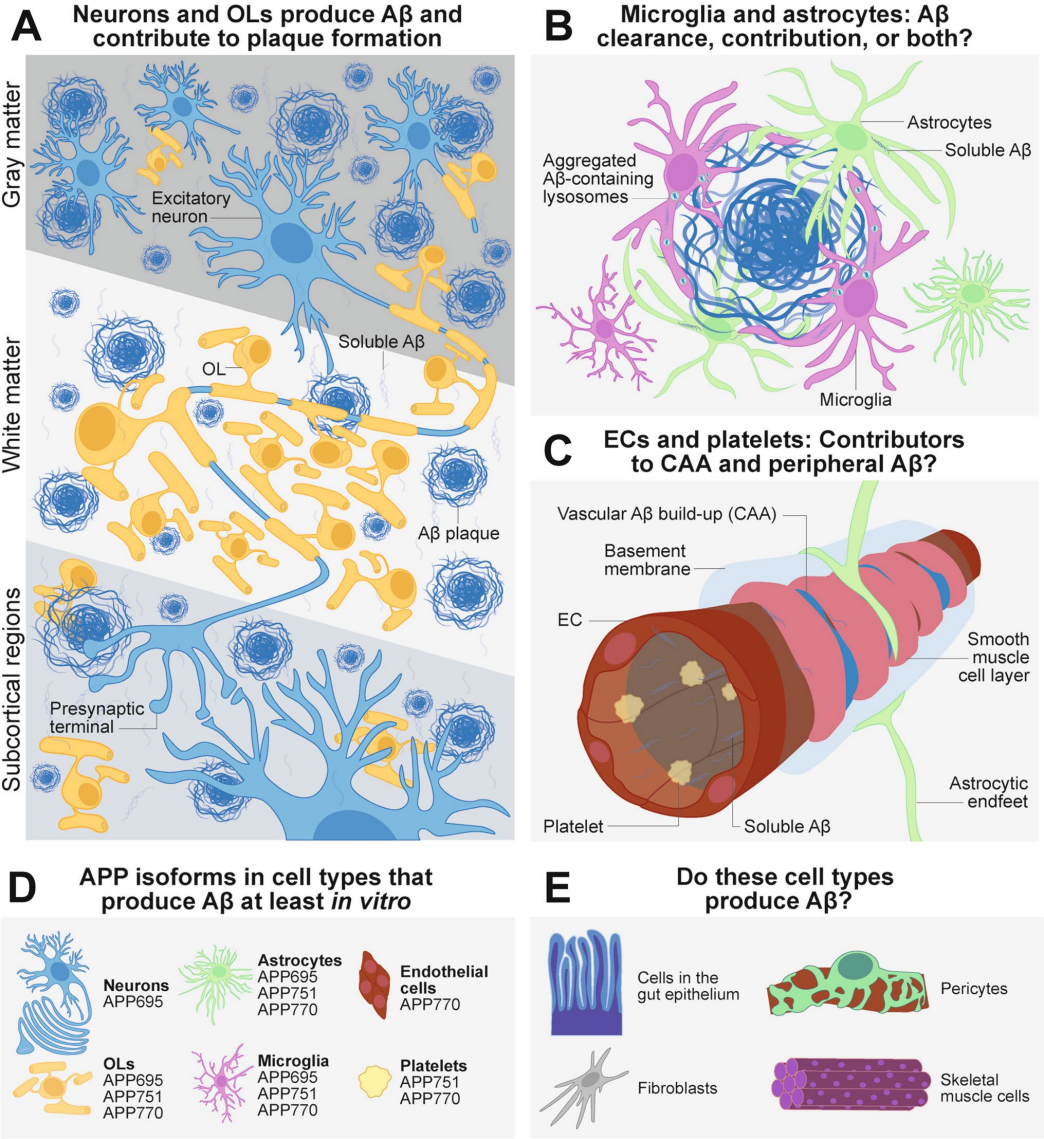
Neuronal and non-neuronal production of Aβ: An ongoing investigation.** A** Excitatory neurons in the dorsal telencephalon produce the majority of Aβ that get deposited as plaques, especially in the gray matter and subcortical regions. Neurons also contribute to plaque deposition in the white matter via Aβ released from long-range axonal projections, based on *in vivo* experimental findings. OLs further contribute to additional Aβ load that forms plaques, especially in the white matter, where OLs predominate. **B** Microglia and astrocytes commonly observed corralling Aβ plaques and exhibiting a reactive phenotype may be involved in both the degradation/clearance of Aβ, as well as producing more Aβ themselves. Aβ plaque compaction has been shown to require APOE in the microglial endolysosomal system. The interaction between Aβ plaques, microglia, and astrocytes may suggest a vicious cycle of worsening amyloidosis and neuroinflammation. **C** ECs may contribute to vascular Aβ deposits in CAA, and platelet Aβ production may account for a significant amount of peripheral Aβ found in the bloodstream. Both cell types may synergize in contributing to both vascular and blood Aβ. **D** Different APP isoforms are predominantly expressed by cell types discussed in this review, based on existing literature. **E** Additional cell types without concrete experimental evidence of Aβ production, but may be further investigated due to the expression of amyloidogenic components or the presence of Aβ deposits in organs/systems where these cell types are abundant.

The following subsections detail current evidence showcasing non-neuronal sources of Aβ, further highlighted in Fig. 6 at the end of the section. It is to be noted here that Aβ production has additionally been considered in other cell types that may not be discussed in the sub-sections below, including adipocytes [126], shown to produce serum amyloid P that induces accumulation of Aβ deposits in the bone marrow [127], as well as fibroblasts and keratinocytes [128]. For this review, only CNS non-neuronal cells (i.e., OLs, microglia, astrocytes, ECs) and platelets, which may have conventional implications for Aβ plaque formation and CAA, will be discussed.

### Oligodendrocytes

OLs, the myelinating glia of the CNS, express APP and its processing components, including BACE and PSEN, at a level comparable to or even higher compared to neurons [82, 105]. In fact, OLs are the only human cell type enriched for *APP, BACE1,* and *PSEN1* shown in The Human Protein Atlas (Fig. 4). *In vitro* experiments highlighted the ability of rat and mouse OLs to produce a considerable amount of Aβ [129, 130] and various isoforms of APP [131]. One enzyme less widely studied that could mediate amyloidogenic processing in OLs is ADAMTS4. It was shown that OLs harvested from mice lacking *Adamts4* fail to produce the N-terminally truncated Aβ species, Aβ4-40, *in vitro* [130].

Recently, we and other research groups conducted separate mouse studies and showed that OLs produce Aβ and contribute to plaque burden. This was achieved by silencing the rate-limiting enzyme in amyloidogenic processing, BACE1, in OLs [82, 100, 132]. Importantly, these studies came to the conclusion that OLs contribute to about a third of cerebral Aβ plaques, although different OL-lineage *Cre* drivers were used to selectively knock out *Bace1* [121, 133], specifically: *Olig2-Cre,* which is a pan-OL lineage marker produced even in OL precursor cells (OPCs) [132], *Cnp-Cre* to target mature OLs [82], and *Plp-CreERT2* to target mature OLs specifically upon tamoxifen induction after developmental myelination has largely ceased [100]. Moreover, it also remains possible that OPCs, also targeted by *Olig2-Cre*, contribute to Aβ load even without differentiating to OLs, as it was shown that cultured rat OPCs are able to secrete Aβ40 and Aβ42, albeit less than OLs, into the culture media [134]. Although all three studies concluded that OLs contribute to the formation of approximately 30% of Aβ plaques in the gray matter, soluble Aβ measurements indicate that OLs may produce 10%–15% of total cortical Aβ, which then aggregates with neuronally derived Aβ [82].

The OL contribution to Aβ plaques is higher in white matter tracts where OLs predominate and myelinate dense clusters of axons [82], further reflecting regional differences in OL to neuron ratio [135], which is also distinct across various species due to differences in brain size and complexity [136]. Notably, one of the starkest differences between the human and mouse brain architectures is the much higher white to gray matter ratio in the human compared to the mouse brain. An ultrastructural characterization of 1 mm^3^ of a human cortical sample also revealed that OLs are the predominant cell type, even in comparison to neurons [137], with OL density gradually increasing approaching the white matter. Thus, this begs the question of whether the OL or glial contribution to Aβ load in humans differs significantly compared to observations made in mouse models. One of the major points of contention about these findings is that Aβ plaques are less frequently seen in the white matter of AD patients. Soluble Aβ levels, however, are increased in the white matter of AD patients [138]. Although this requires further investigation, it is important to note that visualizable Aβ deposits are just one of the many forms of Aβ, and soluble oligomeric Aβ could still accumulate and exert detrimental effects in the white matter, especially if the threshold concentration for Aβ plaque seeding is not reached.

Similar Aβ production and trafficking mechanisms might occur in both OLs and neurons, and they may not be confined to the cell soma. Just as axons and dendrites feature satellite Golgi outposts that could serve as local Aβ production sites [139], satellite Golgi outposts present in OL processes may also support local Aβ generation [140, 141]. How Aβ is released from OLs to the extracellular milieu, however, remains largely unknown—just as Aβ release mechanisms in neurons remain debated—and could involve exosomes [56], which are abundantly secreted by OLs [142]. In conclusion, OLs have recently been shown to produce significant levels of Aβ, which contributes to total Aβ plaque burden. Further functional *in vivo* studies could elucidate whether the benefits of silencing OL Aβ production by selectively targeting *Bace1*, which includes amelioration of neuronal hyperactivity [100], could be further translated and serve as a foundation for novel anti-amyloid therapeutic strategies.

### Microglia

As the tissue-resident macrophage population of the CNS, microglia have been extensively studied in the context of neuroinflammation [143]. In AD mouse models, they have been described to acquire a disease-associated microglial (DAM) transcriptional state [89] and are associated with Aβ plaques [144, 145]. The potential role of microglia in Aβ production might be overshadowed by their role in Aβ removal or even plaque accumulation and seeding. Several mouse studies consistently showed that microglia are essential for plaque deposition [146–148]. Additionally, microglia have also been implicated in spreading Aβ and tau seeds in the context of AD *in vivo* [120, 149].

From transcriptomic datasets, microglia have been shown to exhibit limited expression of amyloidogenic processing machinery [82, 100]. However, one study showed that cultured microglia from APPswe mice secrete detectable levels of Aβ42, which was diminished when treated with a γ-secretase inhibitor [102]. Microglia cultured in the presence of Aβ25-35 or lipopolysaccharide were shown to secrete more Aβ [117], which further hints that APP processing might be regulated differently in various microglial states. Microglia cultured from chick embryos were shown to produce less Aβ1-40 but a higher N-terminally truncated Aβ2-40:Aβ1-40 ratio compared to neurons [150]. The presence of Aβ could also cyclically induce a microglial reaction, which could spark their own amyloidogenic processing.

Importantly, Singh and colleagues assessed 5xFAD mice with a tamoxifen-induced microglial *Bace1* ablation and reported significantly fewer Aβ plaque loads due to enhanced Aβ clearance but retained β-CTF abundance [151], highlighting a potential microglial-specific function of BACE1 in modulating the lysosomal-mediated degradation pathway. Importantly, as 5xFAD mice were used, Singh and colleagues could not assess the potential Aβ plaque contribution from microglia due to the 5xFAD transgene only being expressed in a subset of excitatory neurons. Thus, efforts to selectively silence microglial *Bace1* or other components of the amyloidogenic pathway in *APP*^*NLGF*^ mice, for instance, could confirm if microglia indeed produce considerable amounts of Aβ. However, analysis of such a mouse mutant could prove difficult given that microglia are also involved in Aβ uptake, a process which needs to be disentangled when studying a cell type’s role in Aβ production and plaque deposition. Regardless of the limited evidence of microglial Aβ production, microglia are intimately involved in plaque seeding, compaction, and Aβ clearance.

### Astrocytes

Commonly known to support neuronal function and respond to CNS injury or disease, astrocytes have been shown to secrete detectable Aβ42 when cultured from APPSwe mice [102]. Over 30 years ago, Busciglio and colleagues reported that cultured fetal rat cortical astrocytes secrete 4.7-fold less Aβ compared to cultured rat neurons [152]. Interestingly, however, cultured human cortical astrocytes secrete 3- to 4-fold more Aβ compared to cultured rat cortical astrocytes [152], hinting at cell-type-specific species differences in Aβ production at least *in vitro*. This was corroborated to an extent in a report wherein cultured cortical rat astrocytes secrete Aβ at approximately 25% the amount of Aβ produced by cultured neurons [153]. Interestingly, cultured astrocytes from chick embryos and humans were shown to generate a higher ratio of the N-terminally truncated Aβ Aβ2-40:Aβ1-40 when compared to neurons [150]. The production of Aβ2-40, for example, was relatively unchanged upon administration of BACE inhibitors, indicating that different Aβ isoforms are generated from astrocytes. Zhao and colleagues generated transgenic APP mice driven either by the neuronal NSE or astroglial GFAP promoter, but it was ultimately revealed that astrocytes produce low sAPPβ levels, and Aβ was undetected [96]. Thus, the earlier *in vivo* effort to investigate astroglial Aβ production hinted at the poor Aβ-producing capabilities of astrocytes, if any.

Cultured reactive astrocytes express the amyloidogenic components *APP* and *BACE1* and are capable of producing detectable levels of Aβ40 [154]. The amount of secreted Aβ40 is also elevated upon stimulation with tumor necrosis factor α (TNF-α) and interferon-γ (IFN-γ), as well as treatment with Aβ42. This corroborated earlier observations that primary astrocytes co-stimulated with either IFNγ and TNFα or IFNγ and IL-1β generated higher amounts of Aβ40 and Aβ42 [155].

Much like microglia, however, the potential role of astrocytes in Aβ production is complicated by their function in Aβ degradation and clearance [156, 157]. Upon administration of Aβ42 protofibrils, cultured astrocytes were perplexingly shown to store instead of degrading the ingested Aβ [158]. Aβ was also shown to be robustly taken up by iPSC-derived astrocytes, which resulted in a transition to a reactive cell state when Aβ is stored for an extended period [159]. This cyclically causes the release of inflammatory cytokines and could mediate the reactivity of more astrocytes or microglia. Although microglial APOE has been implicated in plaque compaction [160] and showed elevated release during inflammation [161], astrocytes remain the predominant producers of CNS APOE at baseline [162] with implications for neuronal health [163]. In astrocytes, *APOE4*—the strongest risk factor gene in AD—has been shown to impair the integrity of the BBB [164] and upregulate inflammatory cascades [165].

As inflammatory mediators have been shown to upregulate APP expression [166, 167], it is possible that reactive astrocytes and microglia, releasing ample pro-inflammatory molecules in AD, coexist in a vicious loop of continuously increasing their amyloidogenic component production, leading to a more pronounced Aβ production. Interestingly, different cocktails of pro-inflammatory cytokines have been shown to upregulate amyloidogenic processing components in cultured astrocytes, with the most marked effect being a three-fold increase in APP expression 24 h upon stimulation and a four- to six-fold increase in BACE1 expression 96 h upon stimulation [154]. If astrocytes do in fact produce Aβ, an inflamed environment mediated by reactive microglia is likely to increase astrocytic Aβ production.

### Endothelial Cells

CNS ECs line the inner walls of the blood vessels and are major constituents of the BBB, which limits the brain's exposure to peripheral factors. In the context of AD and in the presence of amyloid pathology, EC integrity is disrupted [168], contributing to BBB dysfunction and leakage. The effects of Aβ on EC have been extensively researched [169–171], especially in the context of CAA [172]. CAA refers to the deposition of Aβ, more frequently Aβ40, primarily on the walls of the leptomeninges and cerebral arteries and arterioles [173]. Notably, CAA is not AD-specific and can be observed in other types of dementia, including Lewy body diseases and Parkinson’s disease [174].

ECs considerably express *APP,* accompanied by the relatively low expression of *BACE1* and *PSEN1* (Fig. 4). ECs predominantly express the APP770 isoform with a higher baseline APP expression in cerebral blood vessels compared to peripheral arteries [175]. Of note, ECs also express similar if not slightly higher levels of *PSEN2* [82]; hence, more studies on EC γ-secretase functions might prove informative. Cultured ECs have been shown to produce Aβ [175], which could have further implications in Aβ generation and deposition in blood vessels in CAA. Hypoxic stress *in vitro* has also been shown to upregulate APP, modulate BACE function, and increase Aβ production from ECs [176, 177]. Similarly to neurons, cultured ECs harboring the APP mutation V717F generated a higher amount of β-CTFs, hinting at elevated amyloidogenic processing [178].

In fact, mice overexpressing wild-type human APP770 specifically in ECs showed elevated serum Aβ and sAPP [179]. Although these mice did not exhibit cortical plaque deposition despite human APP overexpression, a stark CAA exacerbation and a significant increase in parenchymal Aβ plaques were observed upon further crossbreeding with the *APP*^*NLF*^ mice [179], further suggesting that ECs could contribute to cerebrovascular Aβ accumulation. Furthermore, CAA in the double mutants was primarily driven by Aβ40, shown by its increase in the blood vessels, while Aβ42 levels remained stagnant compared to the single mutant controls [179]. This further corroborated the observation wherein Aβ40 is the predominant Aβ species seen in CAA deposits [180]. Although compelling, the study performed by Tachida and colleagues did not conclusively show that ECs produce Aβ and contribute to amyloid burden because of the human APP overexpression component instead of leveraging endogenous murine APP. Conditional deletion of *Bace1* or even *Bace2*, the latter shown to be abundantly expressed in ECs [181], could reveal if ECs produce Aβ *in vivo*. Additional considerations should be taken when discussing the potential role of ECs in generating Aβ, including differences between ECs in the CNS or the BBB and in peripheral blood vessels (Ballabh *et al*., 2004), and why CAA predominantly occurs in the CNS.

### Platelets

The smallest component of blood, platelets, has been reported to abundantly express APP, especially APP770 [182]. As early as 1995, platelets were postulated to be a significant source of Aβ in the blood in a study employing healthy human individuals [183]. In fact, platelets produce Aβ *in vitro* [184], potentially contributing to CAA in AD alongside ECs and the accumulation of Aβ in the bloodstream. Aside from Aβ produced along neuronal projections, Aβ found outside the nervous system could be significantly produced by platelets, which may contribute to peripheral inflammation and worsening neuropathology.

Bu and colleagues conducted parabiosis experiments between 9-month-old APPswe/PS1dE9 transgenic AD mice and their wild-type littermates. In the study, wild-type mice developed histopathological hallmarks of CAA upon analysis at 13 months post-parabiosis as well as elevated tau phosphorylation and neuroinflammation [185]. The development of CAA was attributed to the transfer of circulating Aβ in the bloodstream between AD and wild-type mice [185]. Conclusively, bone marrow cell transplantation from 3-month-old APP/PS1 to age- and sex-matched irradiated wild-type mice resulted in cerebral CAA and Aβ plaque deposition [186], suggesting that blood-derived Aβ—presumably produced by platelets and other circulating cells—is seeding-competent in the CNS. Inversely, bone marrow cell transplantation from wild-type to APP/PS1 mice reduced cerebral Aβ deposition 12 months post-transplantation and ameliorated ensuing neuroinflammation as well as rescued behavioral deficits [186]. Regardless of whether the attenuated Aβ deposition in transplanted APP/PS1 mice is a byproduct of an altered phenotype of immune cells derived from the bone marrow of wild-type mice, this series of studies highlighted the direct implications of peripheral immune cells on Aβ deposition and warrants further investigations into the cell-type-specific sources of Aβ potentially generated in the bloodstream.

A study of AD patient-derived platelets showed elevated Aβ and BACE1 as well as reduced ADAM10 when compared to non-disease controls [187]. Casoli and colleagues additionally showed that platelets—reacting to high-density culture conditions—secrete more Aβ40 into the media than Aβ42 [188]. Similar production of Aβ40 from platelets was also observed upon thrombin or collagen activation of cultured platelets [189]. Roher and colleagues confirmed that platelets from human donors produce 80-fold higher Aβ40 than Aβ42 and that inactivated platelets produce more Aβ compared to activated platelets [190]. Consistent with the discussed potential role of blood- or platelet-derived Aβ in mediating CAA, earlier work described that Aβ40 is the major constituent of Aβ found in CAA plaques [191, 192]. Together with the knowledge that Aβ40 is the predominant Aβ species generated by platelets, it remains a strong possibility that CAA is contributed jointly by cells within the CNS, which also include ECs as well as platelets and potentially other cells within the circulatory system.

To date, the Aβ-producing capabilities of platelets have only been suggested by the many supporting observations in human-derived samples and preclinical studies. Although platelets are unlikely to contribute significantly to cerebral Aβ production and ensuing cerebral plaque burden, platelet-derived Aβ in the blood may represent an overlooked Aβ population with various pathological implications, especially as Aβ has been shown to co-aggregate with various vascular and blood proteins, such as medin [193] and fibrinogen [194], respectively. In fact, Aβ in the blood may act as a chemoattractant and prime peripheral immune cells to home into the brain, where Aβ is abundantly sequestered in the cerebral parenchyma and worsen neuroinflammation, which has been reviewed in greater detail elsewhere [195]. Such a phenomenon may also be aided by the breakdown of the BBB, extensively reported in AD patients [196], which allows ease of entry of peripheral immune cells. Thus, future research should consider platelets as a target to mitigate CAA and circulating Aβ, which may contribute to peripheral immune priming and long-lasting low-grade inflammation.

## Non-neuronal Functions of the APP Processing Machinery

Although APP is perhaps best known as the substrate of Aβ, it is a protein with a multitude of functions that range from cellular adhesion [197] to mediating cellular signaling via its vast network of interactors [198]. Earlier work showed the higher rate of APP production in neurons compared to non-neuronal cells such as astrocytes and microglia [106]. However, with the emerging role of amyloidogenic processing in non-neuronal cells, we also reviewed the functions of APP processing in cell types that are potentially implicated in Aβ production in the subsections below.

### Oligodendrocytes

Why do OLs highly express amyloidogenic components? Similarly to neurons, most APP in OLs resides in the TGN [199]. OLs express all three isoforms of APP, APP695, APP751, and APP770 [131]. Myelination, the process that distinguishes OLs from other cells in the CNS, has been shown to depend on the amyloid processing machinery. One study reported APP immunoreactivity in the rat CNS but not PNS myelin [200]. *App-*deficient mice exhibited failure in remyelination upon cuprizone-mediated demyelination but not wild-type nor *Aplp2*-deficient mice [201]. While *App*-deficient mice showed thinner myelin in the spinal cord, *App-*overexpressors instead showed myelin thickening [202], further highlighting the role that APP plays in modulating myelination. To answer whether myelin changes in *App*-deficient mice are due to the neuronal or OL loss, further work employing cell-type-specific deletion of *App* in both cell types is required.

BACE1 deletion was previously shown to impair CNS, and more prominently, peripheral nervous system (PNS) myelination [203–206] as neuregulin-1—a neuronal protein involved in myelination—is a cleavage substrate of BACE1. Moreover, it was shown that in the CNS, deletion of BACE1 in OLs did not affect myelination profile [82, 132], further suggesting that neuronal but not OL BACE1 exerts effects on myelination [203]. In fact, neuronal neuregulin-1 interacts with the tyrosine kinase, ErbB, on OLs and Schwann cells [207]. Although *Bace1* was conditionally knocked out in neurons via *Thy1-Cre* [203], the effect is impaired PNS but not CNS myelination profile despite the observed reduction in myelin protein expression in the CNS. These findings highlight indirect neuronal-specific effects of BACE1 ablation on other cell types with different implications in OLs and Schwann cells, reflecting distinct mechanisms governing CNS and PNS myelination. On the other hand, γ-secretase inhibition was previously shown to promote differentiation of OPCs—especially when derived from optic nerves—and the myelinating capabilities of OLs in culture [208]. However, mice with a conditional OPC knockout of *Pen-2*, the enhancer constituent of γ-secretase, were shown to develop less mature OLs and recombined OPCs instead differentiated to astrocytes [209], underscoring the potential role of γ-secretase function in OPC cell fate determination via Notch1-independent and -dependent pathways.

The expression of the non-amyloidogenic counterpart of BACE1, the α-secretase A Disintegrin and metalloproteinase domain-containing protein 10 (ADAM10), is reduced by cuprizone-induced demyelination [210]. The same study also showed that overexpression of ADAM10 in the cuprizone model protects against demyelination and attenuates neuroinflammation. In fact, genetic ablation of ADAM10 in OPCs resulted in premature myelination and failure of myelin maintenance, presumably mediated by Notch-1 signaling [211]. Lavaud and colleagues recently reported that OLs sorted from mice with an inducible knock-out of ADAM10 myelinate fewer axons than non-induced controls [212]. When loss of OL ADAM10 was induced in 5-month-old mice, the authors reported a thinning of myelin sheaths 6 months post-induction but, interestingly, a thickening of myelin sheaths 12 months post-induction in the cerebellum [212]. Hence, ADAM10 might also play a role in myelin biogenesis. The ultrastructural myelin change was also met with an increased MBP expression in the cerebellum and the spinal cord at 12 months. Of note, the authors only analyzed mice lacking OL ADAM10 at 12 months but not at 6 months, the latter exhibiting thinner myelin sheaths [212]. Inducible loss of OL ADAM10 also caused impairments in motor functions assessed via the balance beam and accelerating rotarod test [212]. Ultimately, the functions of APP and its processing components in OLs require further research prior to leveraging OL-specific targeting of APP processing as a potential avenue in mitigating amyloidosis in AD.

### Microglia

Although microglial Aβ production remains sparsely considered, various studies have reported functions of APP processing components specific to microglia. Earlier work highlighted the presence of transcripts for the three major APP isoforms, APP695, APP751, and APP770, in cultured rat microglia [213]. Murine *App*-deficient mice infused with oligomeric Aβ displayed attenuated microgliosis [214]. The abrogated microglial reactivity was replicated in APPPS1 mice devoid of their endogenous mouse APP, which also resulted in a markedly reduced Aβ plaque burden [214], highlighting the potential role of APP in mediating reactive microglial states and clearance mechanisms. Prior work has, in fact, shown that APP could act as a proinflammatory receptor in microglia [215]. Moreover, a series of studies showed that administration of sAPPα or sAPPβ on cultured microglia resulted in non-cell autonomous microglial reactivity via Jun N-terminal kinase (JNK) and p38 kinase, resulting in the release of proinflammatory cytokines and reactive oxygen species [216, 217].

A study utilizing iPSC-derived microglia additionally reported that cytokine release in microglia could be mediated by APP and even more potently by β-CTFs [218]. β-CTFs co-assemble with microglial voltage-gated proton channels (Hv1) to form stable complexes, and the presence of several disease-associated APP mutations, such as E682K and D694N, elevated Hv1 current density by approximately six- and 4.5-fold, respectively [218], indicating a heightened cytokine release and neuroinflammation. Hv1 expression is highly microglial-specific in the brain, and loss of Hv1 expression was shown to blunt reactive oxygen species production and proton extrusion, leading to attenuated acidosis [219]. This further highlights the importance of microglial APP and the effects of APP mutations in mediating neuroinflammation [220].

Aside from APP, it was shown that microglial BACE1 is involved in the transition from a homeostatic to a DAM1 signature [221]. Moreover, microglial *Bace1* deficiency enhanced their phagocytic activity, and this effect was replicated by pharmacological inhibition via BACE1 inhibitors [221]. Furthermore, microglial reactivity could be further mediated by Notch signaling [222], which is directly affected by γ-secretase cleavage. By selectively knocking out the gene of a γ-secretase subunit, *Aph1,* in microglia, Hou and colleagues reported upregulation of various DAM genes, including *Timp2*, *Ctsb*, and *Lyz2* [35]. Although selective microglial knockout of *Aph1* did not result in significant cell state changes when mice were bred on a wild-type background, microglial-specific knockout of *Aph1* on an *APP*^*NLGF*^ background revealed that deficient γ-secretase activity resulted in similar transcriptomic changes, which here impeded microglial reactivity. In conclusion, microglial APP processing likely plays a role in mediating reactive state transition and inflammatory profiles.

### Astrocytes

Astrocytic APP expression has been confirmed at least *in vitro* [223]. Much like microglia, Haass and colleagues showed robust expression of transcripts of APP695, APP751, and APP750 in cultured astrocytes [213]. Treatment of cultured astrocytes with transforming growth factor β-1 (TGFβ-1) and interleukin-1 (IL-1) was shown to increase APP expression, especially APP751 and APP770 [224]. APP overexpression in primary human cortical astrocytes treated with lipopolysaccharide (LPS) resulted in a higher production of IFN-γ, IL-10, and IL-13, while reducing IL-6 and TGFβ-1 [225]. These effects were also accompanied by the remodeling of the astroglial intermediate filament, GFAP [225]. Following traumatic brain injury in rodent models, APP and IFN-γ are shown to be elevated in reactive astrocytes. However, in the absence of astroglial APP, LPS-treated astrocytes seem to fail to mount a reactive and inflammatory response [225], further suggesting the astroglial APP-specific functions in the context of neuroinflammation in AD.

Cultured rat astrocytes were shown to express *Bace1* mRNA but not protein [153]. In fact, the same study also reported that BACE2 but not BACE1 is the primary β-cleavage enzyme in astrocytes. In the human brain, BACE2 is abundant, but its activity does not correlate well with Aβ concentration, unlike BACE1 [226]. Hence, it is possible that the steady-state rate of astrocytic Aβ production differs from BACE1-expressing cells if astrocytes do produce Aβ via BACE2.

The Yan lab also investigated the *in vivo* role of astrocyte-specific BACE1 functions on the 5xFAD background [227]. Although this setup did not permit direct inferences on astrocytic Aβ production, Zhou and colleagues found that 5xFAD mice lacking astrocyte-specific *Bace1* developed significantly fewer Aβ plaques [227]. Similar to their microglial *Bace1* ablation study [151], astrocytes cultured from global *Bace1* knockout mice displayed faster Aβ uptake and degradation. This enhanced kinetics was attributed to an upregulation in clusterin—otherwise known as apolipoprotein J—which was previously shown to ameliorate synaptic deficits upon its astrocyte-specific overexpression in 5xFAD mice [228].

Although ADAM10 expression in the context of APP processing is often associated with a beneficial reduction of Aβ production, ADAM10’s role in astrocytes remains understudied. The astrocytic NLRP3 inflammasome was reported to be ADAM10-dependent [229]. The administration of an ADAM10 inhibitor on IL-1β, TNF-α, or IFN-γ-treated human astrocytes resulted in an attenuated shedding of fractalkine (CX3CL1) [230] with presumed downstream effects, which include less microglial CX3CR1 activation and thus, dampening neuroinflammation. During development, however, a lack of CX3CL1-CX3CR1 signaling may delay neurodevelopmental checkpoints and synaptic pruning [231], which further underscores the disease- and developmental-specific context of the functions of ADAM10 and other protease subunits in non-neuronal cells, including astrocytes.

### Endothelial Cells

Physiologically, APP is deemed essential for early angiogenesis [232] and cerebrovascular remodeling upon pathological insults [233] based on *App*-null mouse studies. APP could also play a role in maintaining the tight BBB via ECs, considering reports on APP functions on cell adhesion [234]; however, more specific studies selectively targeting EC APP are required to elucidate the EC-specific functions of the protein. Similar to astrocytes, cultured ECs also respond to inflammatory molecules such as IL-1 by upregulating APP [235]. APP is consistently shown to be upregulated during various forms of stress, potentially as a homeostatic response in ECs [176, 177, 236]. In fact, accumulation of fibrillar Aβ42 could serve as a form of stress, which was shown to enhance Aβ40 and APP production from cultured ECs [237]. EC BACE2 was reported to maintain endothelial homeostasis by preserving nitric oxide synthase functions [181], which worsens oxidative stress. γ-secretase was additionally reported to regulate EC function via vascular endothelial growth factor receptor (VEGFR) cleavage [238]. iPSC-derived ECs from FAD patients harboring *PSEN1* and *PSEN2* mutations revealed impaired tight junction integrity, barrier function, and glucose metabolism, with stronger effects exhibited by *PSEN1* mutant ECs [239].

### Platelets

Although *App*-null mice exhibit unchanged platelet activation, secretion, or aggregation, venous thrombosis was more pronounced in the *App*-deficient mice [240]. To limit the effects of APP-deficiency to blood cells and primarily platelets, Canobbio and colleagues transplanted the bone marrow of *App-*null mice to wild-type mice and still observed worsening of venous thrombosis. The authors then concluded that fibrin formation is negatively modulated by APP on the platelet surface, and potentially due to platelet activity in mediating the formation of neutrophil extracellular trap [240]. α-secratase abundance in platelets was previously shown to be positively correlated with healthy aging [241] and negatively in AD patients [242]. In contrast, a study showed a limited increase of platelet BACE1 in patients with mild cognitive impairment (MCI) [243], while another study employing AD and MCI patients did not report changes in either platelet α-secretase or BACE activity [244]. Conversely, platelet-derived growth factor was reported to activate β- and γ-cleavage in APP-Gal4 HeLa cells [245].

## Targeting Non-neuronal Aβ to Prevent Threshold Concentration for Plaque Seeding

Widespread *Bace1* deletion during adulthood has been shown to markedly reduce existing Aβ plaque deposition and improve cognition *in vivo* [121]. Past clinical trials employing global secretase inhibition resulted in severe adverse effects likely mediated by neuronal toxicity [37, 39]; thus, the question remains if silencing amyloidogenic elements in non-neuronal cell types is a viable option to achieve therapeutically relevant reduction of Aβ levels and plaques. Suppressing Aβ production from OLs rescues abnormal neuronal hyperactivity *in vivo* [100]*,* making it a valuable potential target in AD therapy. Small interfering RNA packaged in nanocomplexes to target specific cell types [246] and other gene therapy strategies [247], for example, could be further investigated when targeting non-neuronal cells. Notably, the OL contribution to Aβ load seen in the aforementioned rodent studies seems to pale in comparison to the neuronal contribution [82, 100]. However, human-derived OLs produce Aβ at the same level [105] if not more [100] than neurons. Thus, the smaller Aβ contribution from OLs is likely caused by the much fewer OLs present in the mouse brain compared to the human brain [135, 137], hinting at a potentially higher contribution of OLs to total Aβ load in humans. Additionally, it remains possible that Aβ processing, localization, and release differ between neurons and non-neuronal cells.

Prior to targeting OLs and other non-neuronal cells in AD, initial findings should be supplemented with research on APP processing in said cell types. Critical research gaps that still need to be addressed include why these proteins are highly expressed, the function of APP processing machinery [211], and the consequences of tampering with such processes in non-neuronal cells. As confirmatory evidence of cell-type-specific production of Aβ remains currently limited to rodent and *in vitro* studies, future research should consider methodologies to dissect cellular sources of Aβ in human cases.

Furthermore, *in vitro* evidence of non-neuronal Aβ production could also simply reflect physiological Aβ production [248] that might not contribute to amyloidosis. For example, Kamenetz and colleagues proposed that Aβ—especially at low concentrations—could help mitigate neuronal hyperactivity and excitotoxicity [97]. Early work by Wujek and colleagues also showed that when cultured in flasks with a surface containing droplets of Aβ fibrils, neurons preferentially grow on these substrate-bound fibrils, unlike neurons cultured with Aβ in culture medium suspension [249]. Additionally, secretase inhibitors induce neuronal death *in vitro*, and administration of Aβ—especially Aβ1-40—is protective against this loss of neuronal viability [250]. Aβ was also shown to protect against metal toxicity [251] as metal ion binding in turn increases Aβ aggregation [252, 253], oxidative stress [254], or microbial infection [255]. Although the physiological functions of Aβ could easily be deemed protective, it is important to note that the protection conferred by Aβ could result in the survival of Aβ-producing cells that could be sustained over a long period, eventually also resulting in cerebral amyloidosis.

Finally, shifting strategies from global inhibition of genetic components to selective inhibition or allosteric modulation [256] could prove beneficial. Overexpression of ADAM10 in transgenic APP(V717I) mice resulted in attenuated Aβ plaque deposition, while the overexpression of a catalytically inactive ADAM10 in the same transgenic model exacerbated plaque load [257]. Given the seemingly competitive nature between α- and β-cleavage in APP processing, α-secretase enhancers [258] could serve as another therapeutic avenue.

## Conclusion and Future Perspectives

Although neurons produce the majority of Aβ *in vivo*, future studies should also consider the importance of non-neuronal cells in the pathogenesis of cerebral amyloidosis. Differences in expression of APP isoform between cell types may also result in distinct production rates of various Aβ species and may explain why certain cell types express more Aβ than others [259]. Recent studies that showcased the contributory role of OLs, a glial cell type—even in establishing amyloidosis in AD—highlight how, in most diseases, no cell type is likely to bear sole responsibility in disease pathogenesis. Conceptually, white matter tracts could be a region at high risk for damage due to soluble Aβ, especially considering the contribution of OLs to Aβ load *in vivo* is contrasted with the limited white matter plaques seen in human AD cases [138]. Additionally, total Aβ concentration in AD patient gray matter was observed to be only threefold higher compared to the white matter Aβ concentration [190], highlighting the abundance of Aβ in a brain region not canonically known to exhibit major plaque deposition.

Due to neuroanatomical and neural cell type proportional differences, *in vivo* research should also expand investigations into Aβ production or its processing components from non-neuronal cells in other disease models, be it aged or genetically modified larger mammals, including non-human primates with a neuroanatomical composition more akin to humans than rodent models [260–263]. Moreover, persisting questions remain on the processing and release of Aβ, which may differ depending on the cell type, be it their localization, size, or local density. These investigations may be supplemented with postmortem human sample analysis via multi-omics, *in situ* hybridization, or immunolabeling approaches to further dissect cell types that express the canonical amyloidogenic components. For instance, various publicly available single-cell or spatial transcriptomic datasets show high expression of Aβ processing components in human samples [264–267], which should catalyze a growing interest in the role of non-neuronal cells in AD. Such studies should also be accompanied by equally rigorous efforts to understand the non-Aβ-related roles of APP processing proteins that are expressed in non-neuronal cells.

Conceptually, it is possible that worsening cellular phenotypes additionally trigger microenvironments that preferentially produce Aβ [268], further expediting disease progression. Studies investigating the potential microglial or astrocytic Aβ production are complicated by the role of these cell types in promoting Aβ clearance and plaque formation. On the other hand, EC or platelet Aβ production measurements could prove difficult due to their proximity to the vasculature. ECs might instead play a significant role in mediating CAA, while platelets may contribute to additional Aβ production in the periphery. In fact, peripheral Aβ deposition may be relevant to the disease, given Aβ’s propensity to co-aggregate with vascular proteins such as medin [193] and blood proteins such as fibrinogen [194], which may impair Aβ clearance. Given that clearance of Aβ from the CNS worsens as AD progresses [269], pinpointing the exact amount of Aβ contribution per cell type is precarious due to potential differences along disease progression. Additionally, the co-aggregation propensity of Aβ with other molecules in different organs and systems implies body-wide differences in the effects of Aβ deposition, with further implications and potentially different sets of challenges to effectively remove Aβ outside the brain. Approaches to prevent the threshold concentration of Aβ from being reached for plaque deposition by leveraging non-neuronal sources of Aβ could prove beneficial in developing AD therapeutic strategies, especially when detrimental physiological effects could be avoided.
